# Poster Presentations - Abstracts of the 16^th^ REDLARA Taller General, Medellin - Colombia, 27-30 April 2023

**DOI:** 10.5935/1518-0557.20230020

**Published:** 2023

**P-16. A new semen processing protocol for high sperm DNA fragmentation index samples using microfluidic devices**

**Luiza Mezzomo Donatti^1^, Simone Mattiello^2^, Norma Pagnoncelli Oliveira^1^, Caroline Gross Dutra^1^, Carolina Lumertz Martello^1^, Gabriella Mamede Andrade^1^, Marília Körbes Rockenbach^1^, Nilo Frantz^2^**

^1^Nilo Frantz Medicina Reprodutiva - Embryology, Porto Alegre, RS, Brazil.

^2^Nilo Frantz Medicina Reprodutiva - Clinician, Porto Alegre, RS, Brazil.

**Introduction:** High sperm DNA fragmentation index (sDFI) is associated with lower blastocyst formation and decreased in vitro fertilization (IVF) outcomes (Bajaj & Kapoor, 2022). Centrifugations during semen processing seem to accentuate sDFI while centrifugation-free protocols, as microfluidic sperm-sorting devices, may overcome these disadvantages and select spermatozoa with lower DFI (Ataei *et al*., 2021; Gode *et al*., 2019).

**Case Report:** A 38y old couple was submitted to infertility treatment due to asthenozoospermia and 62% sDFI. The high sDFI treatment with antioxidants and lifestyle changes was suggested but was not performed. The first IVF cycle was carried out with GnRH antagonist protocol and u-FSH + u-LH combined with r-FSH were used for ovarian stimulation. Oocyte pickup (OPU) was performed on the 12th day of the cycle, 36h after triggering with 10.000 UI hCG + 20 UI GnRH analogue. Sperm were collected by masturbation after 48h of sexual abstinence and processed by density gradient protocol. From OPU, 13 oocytes were retrieved and incubated in continuous single culture medium supplemented with 10% serum substitute supplement (Irvine Scientific, California - USA) for 3h. After denudation, oocytes were classified according to the maturation stage: 10 metaphase II oocytes (M2), 1 metaphase I oocyte, and 2 germinal vesicle stage. The 10 M2 oocytes were inseminated by intracytoplasmic sperm injection (ICSI) and after 18h, 8 zygotes presented normal fertilization with 2 pronuclei (2PN). Embryos were cultivated from D0 until D7 under low oxygen (5%) conditions in continuous single culture medium complete (Irvine Scientific, California - USA). Blastocysts were classified according to Gardner and Schoolcraft’s scoring system and good-quality blastocysts were biopsied (Gardner *et al*., 2000). Two good-quality blastocysts were biopsied and submitted to genetic analysis: the D5-blastocyst was diagnosed as aneuploid whereas the D6-blastocyst was euploid. Frozen embryo transfer (FET) was performed 5 days after ovulation during a natural cycle of endometrium preparation. The endometrium was 9mm in thickness. At the transfer, the thawed D6-blastocyst presented an initial hatching. A positive beta-hCG was obtained 10 days after FET. There was a gestational sac at 6-week echography, but a 7-weeks pregnancy loss occurred. The couple returned to a new IVF cycle and the ovarian stimulation protocol was similar to first one. OPU was performed 36h after administration of 40 UI GnRH analogue. Considering the high sDFI, a new semen processing protocol was tested. A serial ejaculation was performed starting a week before the OPU day, with ejaculations every other day, and the sample used for ICSI was collected after 24h of sexual abstinence. The sperm sample was processed with a microfluidic sperm-sorting device (Zymot, Maryland - USA) following the manufacturer’s protocol. From 18 oocytes retrieved, 14 M2 were submitted to ICSI. Embryo culture followed the same laboratory protocols. At D1 stage 12 zygotes were 2PN and 7 good-quality blastocysts were biopsied between D5 and D6 stage. One D5-blastocyst and two D6-blastocysts were diagnosed as euploid. An artificial endometrial preparation was made with 6mg of estradiol daily. The endometrium reached 8.5mm of thickness. On the 14th day was started a supplementation with intravaginal progesterone (800mg/day) and oral dydrogesterone (20mg/day). The thawed D5-blastocyst was expanded at transfer time. Beta-hCG was positive on the 10th day after FET (253 mUI/mL) and 6-week echography confirmed a single gestational sac and heartbeat. The patient is currently in the third trimester of pregnancy at the time of writing. This protocol for high sDFI, associating serial ejaculation and microfluidic device, improved the blastocyst formation and, consequently, the chance of a positive outcome.

**REFERENCES**

Ataei A, Lau AWC, Asghar W. A microfluidic sperm-sorting device based on rheotaxis effect. Microfluid Nanofluidics. 2021;25:52. DOI: 10.1007/s10404-021-02453-8

Bajaj B, Kapoor G. Sperm DNA fragmentation and reproductive outcomes. Fertil Sci Res. 2022;9:10-5. DOI: 10.4103/fsr.fsr_49_21

Gardner DK, Lane M, Stevens J, Schlenker T, Schoolcraft WB. Blastocyst score affects implantation and pregnancy outcome: towards a single blastocyst transfer. Fertil Steril. 2000;73:1155-8. PMID: 10856474 DOI: /10.1016/S0015-0282(00)00518-5

Gode F, Bodur T, Gunturkun F, Gurbuz AS, Tamer B, Pala I, Isik AZ. Comparison of microfluid sperm sorting chip and density gradient methods for use in intrauterine insemination cycles. Fertil Steril. 2019;112:842-8.e1. PMID: 31543253 DOI: 10.1016/j.fertnstert.2019.06.037

**P-01. Development of a predictive model of embryonic ploidy based on the KIDSCORE and iDASCORE algorithms**

**Fernando Peña Espinoza^1^, Eduardo Gazzo Benavides^1^, Gigliana Catanzaro Foppiano^1^, Rocio Dávalos Torres^1^, Jose Luis Llanos Carrillo^1^, Mario Ascenzo Battistini^1^, Marcelo Velit Suarez^1^, Luis Ernesto Escudero Velando^1^**

^1^Inmater Fertility Clinic Lima, Peru.

**Introduction:** Fertility in women decreases as their age increases. Due to decreased ovarian reserve as well as impaired oocyte quality leading to increased aneuploidy in embryos. Studies have identified that the main cause of spontaneous abortions is due to the presence of aneuploidies in fetuses (natural causes) or embryos (IVF). Preimplantation genetic diagnosis (PGT) of embryos during in vitro culture represents a convenient tool to reduce the time to get pregnant. The analysis by predictive algorithms obtained by time-lapse culture system represents a great advantage, since it is capable of processing a wide variety of data points on the embryo development associated with implantation. KIDScore is an embryo selection tool that scores embryos according to their statistical viability based on a series of events and morphological characteristics manually annotated by embryologists after observation of all embryonic development during culture by time-lapse. iDAScore doesn’t require the intervention of the embryologist to perform the annotations, as it makes use of deep learning to provide a fully automated analysis of developing embryos.

Objective: To evaluate the performance of a human blastocyst ploidy prediction model based on the KIDScore and iDAScore algorithm.

**Material and Methods:** Retrospective cohort, 2,867 embryos from IVF/ICSI were evaluated at the Inmater Fertility Clinic in 2022. All embryos were cultured in Embryoscope+ incubator and biopsied on day 5 or 6 when they reached the expanded or hatching blastocyst stage. Information was extracted on oocyte age, oocyte origin, biopsy day, morphology according to Gardner criteria, stage classification and ploidy (preimplantation genetic testing for aneuploidy - PGT-a). In addition, the KIDScore was assessed on 626 embryos and the iDAScore on 1,085 embryos. Gardner's morphology criteria were stratified into four grades: excellent: AA; good: AB, BA; fair: BB, Poor: others).

**Results:** Differences were found between the frequencies of ploidy categories between the groups of oocyte origin, biopsy day and morphology (Table 1). Similarly, the average oocyte age, KIDScore and iDAScore are different between aneuploidy and euploid embryos. The adjusted model showed that for each point increase in iDAScore and KIDScore the probability of aneuploidy is reduced by 12% (95%CI 19% - 4%) and 15% (95%CI 26% - 5%) respectively (Table 2). On the other hand, the internal validation of the predictive logistic regression model with the bootstrap technique showed better results (iDAScore c-statistic: 0.67, KIDScore c-statistic: 0.68) (Figure 1) compared to the AUC of the KIDScore (0.36, 95%CI 0.31-0.40) and iDAScore (0.40, 95%CI 0.36-0.43) (Figure 2).

**Table 1:** Clinical and demographic characteristics associated with the presence of aneuploidy.

	Ploidy		*p* value
	Aneuploidy (n=1,175)	Euploidy (n=1,692)	
Oocyte age^*^	31 (±7)	28 (±7)	<0.001
Oocyte origin^†^ Own oocyte Donate oocyte	649 (48.5) 526 (34.4)	689 (51.5) 1,003 (65.6)	<0.001
Biopy Day^†^ Day 5 Day 6	665 (38.7) 510 (44.4)	1,054 (61.3) 638 (55.6)	0.002
Gardner criteria^†^ Excellent Good Fair Poor	265 (30.3) 345 (39.0) 371 (48.2) 174 (62.6)	610 (69.7) 539 (61.0) 398 (51.8) 104 (37.4)	<0.001
Classification^†^ Expanded blastocyst Hatching Blastocyst	26 (44.1) 1,137 (41.1)	33 (55.9) 1,627 (58.9)	0.651
iDAScore score^*^	6.7 (±1.7)	7.3 (±1.6)	<0.001
KIDScore score^*^	6.0 (±1.8)	6.9 (±1.7)	<0.001

^*^Mean, SD. Student’s test

† n, percentage. Chi-square test.

**Table 2:** Crude and adjusted logistic regression model for the presence of aneuploidy.

	OR	CI 95%	*p* value
Crude model			
iDAScore score	0.82	0.76-0.88	<0.001
KIDScore score	0.75	0.68-0.83	<0.001
Oocyte age	1.06	1.05-1.08	<0.001
Gardner criteria Excellent Good Fair Poor	1 1.47 2.15 3.85	Ref 1.21-1.80 1.75-2.63 2.90-5.11	<0.001 <0.001 <0.001
**iDAScore adjusted model^†^**			
iDAScore score	0.88	0.81-0.96	0.003
Oocyte age	1.07	1.05-1.09	<0.001
Gardner criteria Excellent Good Fair Poor	1 1.19 1.65 2.91	Ref 0.84-1.70 1.14-2.40 1.77-4.80	0.331 0.008 0.000
**KIDScore adjusted model^†^**			
KIDScore score	0.85	0.74-0.95	0.006
Oocyte age	1.06	1.03-1.08	<0.001
Gardner criteria Excellent Good Fair Poor	1 1.56 1.94 2.32	Ref 0.99-2.46 1.17-3.21 1.13-4.77	0.055 0.010 0.022

^*^OR: Odds ratio CI 95%: Confidence interval at 95%

† Adjusted logistic regression model based on the stepwise (forward-backward) method.

Note: The overall model is significant according to the model's chi-square statistic. However, the variability of the outcome is explained by the error and not by the model.

**Table 1:** Pathologies identified with diagnostic hysteroscopy.

Pathology	Cases (%)	Age
Endometrial polyps	11.59	37.67±0.67^*^, ^**^
Cervical polyps	7.25	35.40±1.34^**^
Ostia polyps	0.48	33.00
Endometrial polyps + other uterine pathologies	1.93	35.75±1.89
Other uterine pathologies	4.83	36.80±1.41
No pathologies	73.91	34.92±0.45^*^

^*^,

** Statistical difference between group of patients.

**Table 1:** Comparison between niPGT-A and FET groups.

	ni-PGT (n=11)	elective FET (n=15)	tvalue or p-value (95% CI)
**Average age**	36.3±4.4	35.4±4.8	t=1.0606
**Biochemical (βhCG)**	4/11 (36.4%)	5/15 (33.3%)	*p*=0.8725
**Clinical pregnancy/βhCG**	4/4 (100%)	4/5 (80%)	*p*=0.6084

**Table 1:** Age of the patients and the PGT-A results between pre and post PRP cycles.

Patient	Age (y)	PRE PRP Cycle # Euploid Embryos	# Weeks Between Cycles	POST PRP Cycle # Euploid Embryos	% Euploid Embryos Before → After PRP
1	40	0	6	1	0 → 100^*^
2	35	0	8	3	0 → 75^*^
3	40	1	12	3	50 → 75
4	35	0	14	0	0 → 0^*^
5	40	0	6	1	0 → 50^*^
6	38	0	10	0	0 → 0^*^
7	39	1	6	1	50 → 50
8	38	1	16	1	100 → 33.3
9	32	0	6	0	0 → 0^*^
10	36	0	12	0	0 → 0^*^
11	35	0	12	0	0 → 0^*^
12	39	1	8	2	33.3 → 66.7
13	42	1	8	0	33.3 → 0
14	39	0	12	0	0 → 0^*^
15	35	3	10	1	50 → 50
16	41	0	18	0	0 → 0^*^
17	31	0	10	1	0 → 100^*^
18	43	0	12	0	0 → 0^*^
19	43	1	18	0	33.3 → 0
20	41	0	14	0	0 → 0^*^
21	37	0	6	3	0 → 50^*^
22	36	2	6	0	66.7 → 0
23	37	1	6	2	100 → 66.7
24	41	0	6	1	0 → 50^*^
25	35	1	6	2	50 → 50
26	40	0	12	1	0 → 100^*^
27	31	0	6	1	0 → 50^*^
28	40	2	10	0	66.7 → 0
29	43	0	10	0	0 → 0^*^

^*^ Patients who failed to obtain any euploid embryo in the pre-PRP cycle.

**Table 1:** Relationship of the BMI with the proportion of patients with normal or altered %DFI in the 3 age groups evaluated.

Age	BMI	Normal (n=614)	Abnormal (n=236)	*p*-value
**<=30** n=126	**Normal weight** **Overweight** **Obesity**	17 (100%) 65 (83%) 22 (71%)	0 (0%) 13 (17%) 9 (29%)	0.2735
**31-40** n=446	**Normal weight** **Overweight** **Obesity**	46 (77%) 177 (73%) 107 (74%)	14 (23%) 65 (27%) 37 (26%)	0.5072
**>=41** n=278	**Normal weight** **Overweight** **Obesity**	21 (70%) 113 (65%) 46 (63%)	9 (30%) 62 (35%) 27 (37%)	0.7378

**Table 1:** Chi-square test of independence of the age group with the % DFI Normal-Abnormal.

Age	%DFI		*p*-value
	Normal	Abnormal	
<30 (n=200)	170 (85%)	30 (15%)	0.000
31-40 (n=773)	586 (76%)	187 (24%)	
41-50 (n=420)	282 (67%)	138 (33%)	
>50 (n=119)	67 (56%)	52 (44%)	

**Table 1:** Chi-square test of independence of age groups with % Normal-Abnormal DFI in normozoospermic patients.

Age	%DFI		*p*-value
	Normal	Abnormal	
<30 n=159	145 (91%)	14 (9%)	0.000^***^
31-40 n=561	482 (86%)	79 (14%)	
41-50 n= 288	229 (80%)	59 (20%)	
>50 n=75	53 (71%)	22 (29%)	

**Table 1:** Results.

	Correct answer	% of correct answers	% of “don´t know”
Eggs are the same age as women	True	47.5%	20.1%
Nowadays women in her forties have the same chance of pregnancy than a woman in her thirties	False	58.9%	12.9%
The use of oral contraceptive (the pill) for more than 5 years protects women ovarian reserve	False	55.5%	39%
We consider that a couple has fertility problems when they can´t get pregnant after a year of regular sexual intercourse and without using a contraceptive method	True	64.9%	22.1%
About 1 in 10 couples are infertile	True	25.7%	68.4%
A woman that never menstruates is still fertile	False	33.7%	37%
IVF is most of the time the treatment choice in couples with fertility problems	False	62.1%	22.9%
Assisted reproduction techniques allows most women to get pregnant with their own oocytes if they have not entered menopause	False	12.2%	27.7%
In our country, high complexity treatments are subsidized by the National Resource Fund (FNR) regardless of the age of the woman	False	52.6%	32.2%
Low complexity treatments are covered by Health Care Providers	True	26.1%	43.6%
Our national assisted reproduction law provides coverage to woman without a partner and woman-woman couples	True	46.9%	46.8%
There is a progressive decrease in pregnancy rates (lower fertility) in women from the age of 36	True	83.6%	13.4%
Miscarriage rate is significantly higher in women over the age 40	True	57.6%	33.7%
The vitrification of eggs before the age of 35significantly prolongs female fertility.	True	58.1%	29.5%
Sexually transmitted infections (such as chlamydia and gonorrhea) significantly increase the risk of subsequent infertility	True	46.1%	43.1%
If a man produces semen, it means that he is fertile	False	96%	2.9%
IVF impacts negatively impacts my long-term health	False	61.3%	35.1%
Children conceived through highly complex assisted reproduction techniques have greater long-term health problems than naturally conceived children	False	75.8%	21.8%
Difficulties in achieving pregnancy are usually linked to female causes	False	75.4%	14.5%
Most women undergo more than one IVF to achieve pregnancy	True	55%	35%
Having a healthy lifestyle improves women's fertility	False	8.8%	14.7%
Assisted reproduction treatment guarantees pregnancy	False	92.1%	6.1%
If a man has mumps after puberty, it is very likely that he will have fertility problems afterwards	True	29.8%	46.1%
If a man achieves an erection, it is an indicator that he is fertile	False	97.7%	1.9%
Women’s weight affects chances of pregnancy	True	60.6%	19.5%
Smoking negatively affects female fertility	True	73.9%	18.7%
Smoking negatively affects male fertility	True	67.2%	25.1%

**Table 1:** Results regarding imported oocytes to in vitro fertilization.

		variation (min-max), per couple	mean±SD, per couple
**Oocyte (n)** Thawed oocyte oocyte Inseminated Fertilized oocytes Divided oocyte Total number of blastocyst (D5+D6)	849 786 606 574 358	5 to 9 2 to 9 1 to 9 1 to 9 0 to 8	7.47±1.44 7.10±1.64 5.78±1.74 5.55±1.83 3.85±1.72
**Rates (%)** oocyte survival rate (Inseminated/thawed)^*^ Fertilization rate (fertilized/Inseminated) Blastocyst rate (Blastocyst/fertilized)	92.6 77.1 59.1		
**Blastocyst** Dynamic Score - Embryoscope		1.5 to 9.7	4.85±2.09

^*^only one couple had 0% of survival.

**Figure 1:**
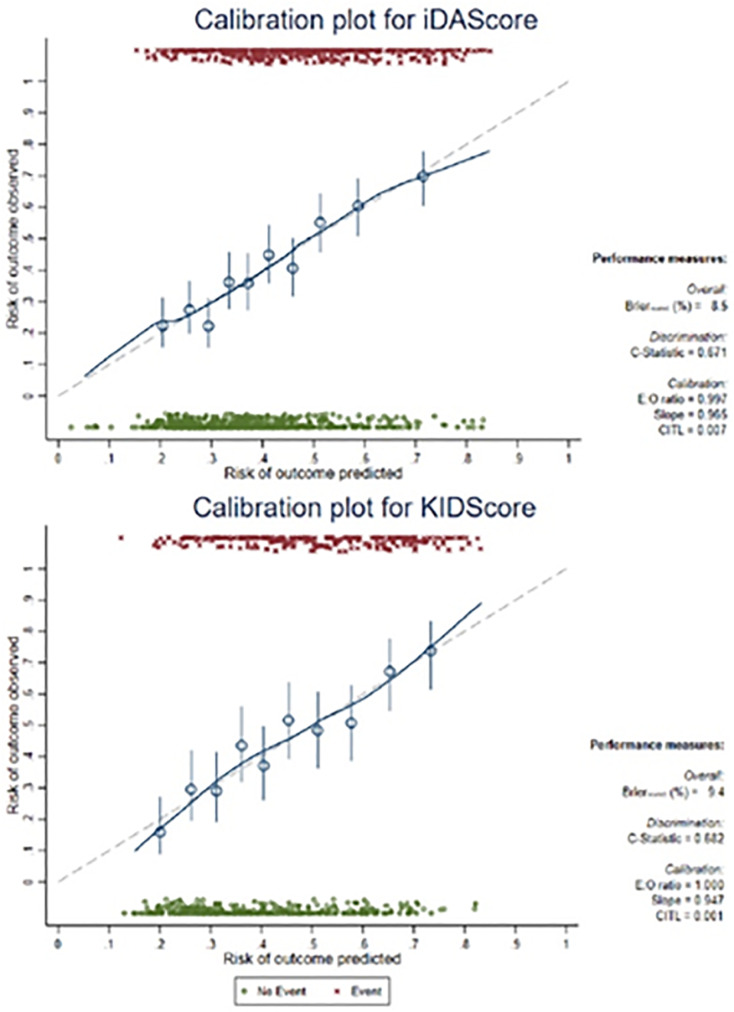
Aneuploidy prediction model calibration plot.

**Figure 2:**
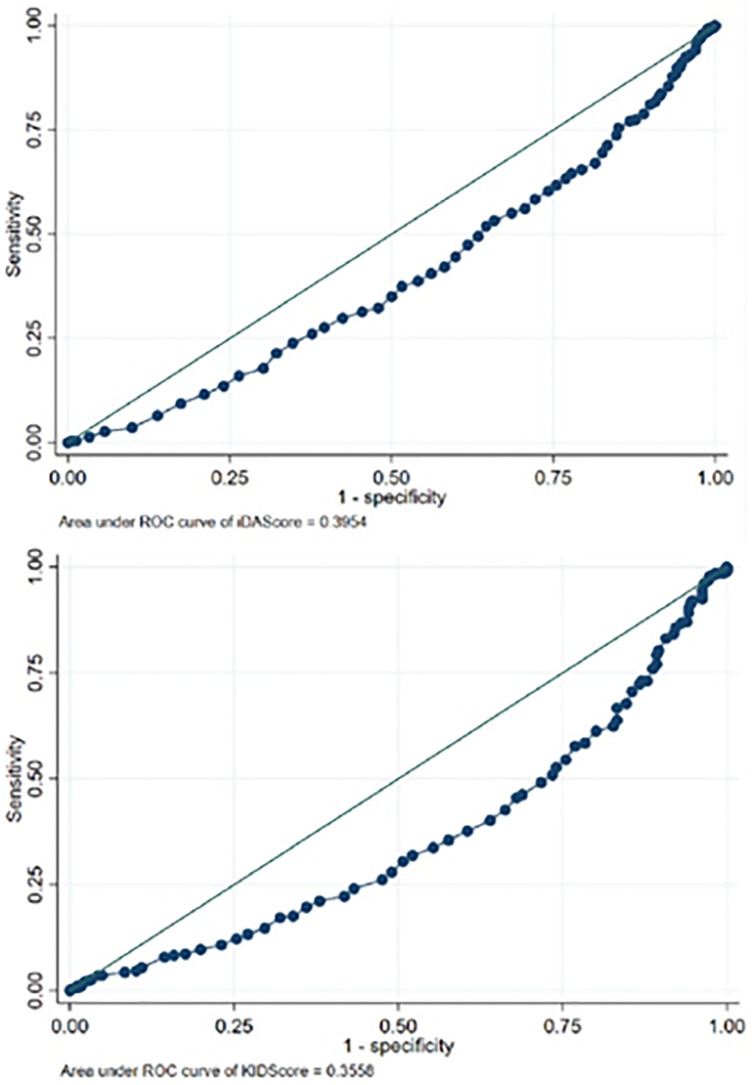
(a) ROC curve (Receiver Operation Characteristic) for aneuploidy prediction from the KIDScore instrument (b) and the iDAScore algorithm.

**Figure 1:**
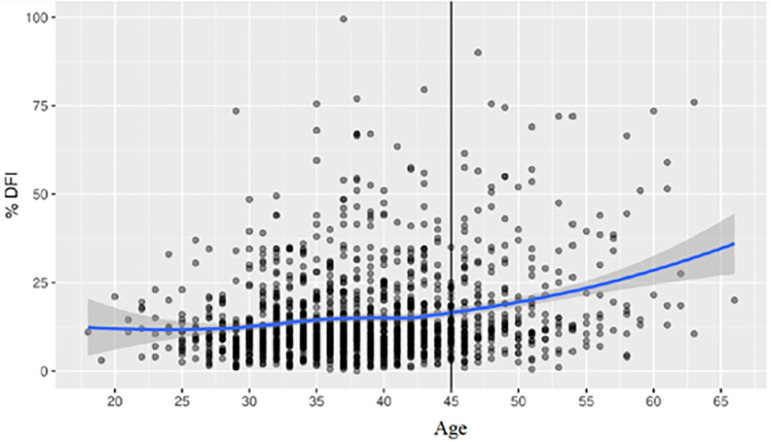
Davies test looking for changes in trend, showing the best cut-off point (*p*-value=0.01245).

**Figure 2:**
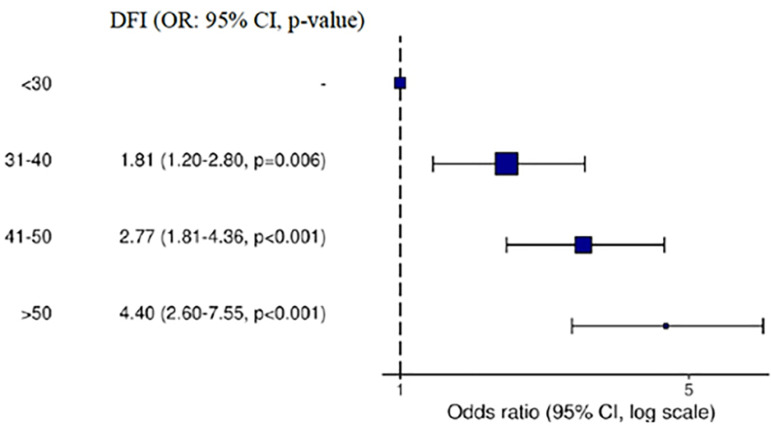
Forest plot showing the odds ratios with their respective confidence intervals in the age groups tested, taking age <30 as a reference.

**Figure 1:**
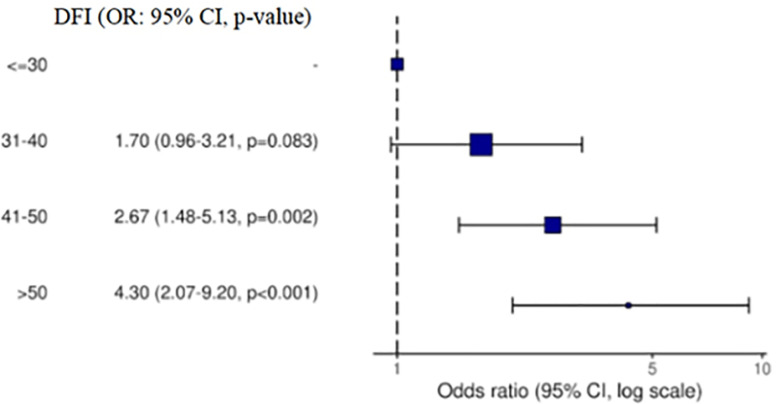
Forest plot of normozoospermic individuals, showing the odds ratios with their respective confidence intervals in the age groups tested, taking age <30 as a reference.

**Conclusions:** The ability of the KIDScore and iDAScore algorithms to predict the ploidy of human blastocysts is very limited. However, when these algorithms are associated with the morphological assessment from Gardner’s criteria and the age of the oocytes, their performance improves substantially. Additionally, there are no significant differences between both types of algorithms, the KIDScore being slightly higher than the iDAScore, which shows us that the intervention of the embryologist, for this type of analysis, is still necessary.

**P-03. Cullen's sign following oocyte pick-up for fertility preservation**

**MEB Amaral^1^, HM Nakagawa^1^, I Cabral^1^, JR Iglesias^1^, ACP Barbosa^1^, AA Silva^1^**

^1^GENESIS-Centre for Assistance in Human Reproduction, Brasília, DF, Brazil.

**Introduction:** Cullen sign is a physical exam finding of ecchymoses around the umbilicus; It is rare, being seen in less than 1% of ectopic pregnancies, and in 1% to 3% of pancreatitis patients (Wright, 2016). Cullen sign is a marker of retroperitoneal hematoma. A previous report of two cases of Cullen sign after ovarian stimulation for IVF in literature was found, in which patients where both high responders (Bentov *et al*., 2006). Here we describe the appearance of the sign in a poor responder patient after a non-eventful oocyte retrieval.

**Case description:** A 36-year-old woman was treated in our clinic for oocyte cryopreservation for social reasons. She had adenomyosis and ASRM’s stage I endometriosis, graded by ultrasound and pelvic MRI. Her ovarian reserve evaluation showed and AFC=09 and AMH=0.43 ng/mL. We proposed to do two sequential oocyte pick-ups. The first stimulation was a progestin primed protocol, using FSHr + FSHr/LHr. Ovulation was triggered with GnRh agonist and 35 hours after the trigger 8 follicles larger than 13 mm were aspirated in an uneventful pick-up procedure. Only 3 oocytes were retrieved. Five days after the patient restarted ovarian stimulation, this time with an antagonist protocol using a mix of FSHr and FSHr/LHr. Ovulation was triggered with a GnRh agonist and uHCG simultaneously 36h before the retrieval. Oocyte retrieval went out without complications, 8 follicles larger than 13 mm were aspirated and 6 oocytes were vitrified. One week after the procedure the patient made contact referring a periumbilical hematoma. She had also mild pelvic pain and abdominal distension. Patient was admitted to perform workup for pancreatitis and coagulopathies, a blood panel, and an abdominal CT scan. All exams where negative for hemorrhage and patient was discharged home. The periumbilical hematoma had spontaneous resolution in one week. **Discussion:** Cullen’s sign represents either retroperitoneal bleeding or hemoperitoneum. It is classically associated with pancreatitis, but the sign has been reported in a variety of conditions such as ectopic pregnancy, perforated duodenal ulcer and intraabdominal non-Hodking lymphoma (Zuin *et al*., 2021; Raw, 1971; Silvestre *et al*., 1996). The bleeding out of follicles can spread between the peritoneal folds to the retroperitoneal space. From there blood could make it way via the falciform ligament to the periumbilical region. The presence of the sign should prompt further investigation. But, as show in this case, the sign is not correlated to the gravity of the underlying cause and can have a benign course.

**REFERENCES**

Bentov Y, Levitas E, Silberstein T, Potashnik G. Cullen's sign following ultrasound-guided transvaginal oocyte retrieval. Fertil Steril. 2006;85:227. PMID: 16412762 DOI: 10.1016/j.fertnstert.2005.06.054

Raw SC. Cullen's sign in perforated duodenal ulcer. Br Med J. 1971;1:505. PMID: 5544017 DOI: 10.1136/bmj.1.5747.505

Silvestre JF, Jover R, Betlloch I, Carnicer F, Bañuls J, Pérez-Mateo M. Cullen's sign secondary to intra-abdominal non-Hodgkin's lymphoma. Am J Gastroenterol. 1996;91:1040-1. PMID: 8633549

Wright W. Cullen Sign and Grey Turner Sign Revisited. J Am Osteopath Assoc. 2016;116:398-401. DOI: 10.7556/jaoa.2016.081

Zuin M, Zuliani G, Rigatelli G, Roncon L. Cullen's sign associated with ectopic pregnancy. QJM. 2021;114:423. DOI: 10.1093/qjmed/hcab040

**P-02. iDAScore, MitoScore and Endometrial thickness as predictive tools for implantation rate in euploid embryos from patients undergoing assisted reproductive procedures**

**Eduardo Gazzo Benavides^1^, Fernando Peña^1^, Rocio Dávalos^1^, Jose Luis Llanos^1^, Gigliana Catanzaro^1^, Mario Ascenzo^1^, Marcelo Velit^1^, Ernesto Escudero^1^**

^1^Clínica Inmater, Peru.

**Introduction:** The current goal of assistive reproduction is to achieve pregnancy with a healthy baby after the first embryo transfer. For this purpose, reproduction laboratories should use all available selection tools. Genetic diagnostic for aneuploidy is a widely used tool, usually the first choice for embryo selection to identify euploid embryos. The quantification of mitochondrial DNA within the embryo is known as the MitoScore (Igenomix). Initial research on the MitoScore revealed that higher mtDNA in euploid embryos is associated with poor implantation potential and could indicate reduced metabolic fuel during oocyte maturation. Embryo selection using Time Lapse technology and algorithms has also become a frequently used tool, one such embryo selection algorithm is the iDAScore from Vitrolife. Some work has reported that it could be helpful in selecting the best embryo from a cohort of embryos. Finally, we have endometrial thickness as an indicator of endometrial receptivity, with publications reporting associations between live birth rates and endometrial thickness.

**Material and Methods:** In this paper, we want to evaluate the predictive capacity of the iDAScore for embryo implantation. In addition, to study whether there is an association between the values of iDAScore, MitoScore, and endometrial thickness with implantation rates, considering maternal age, Gardner criteria, and the day of embryo biopsy. A retrospective cohort study included 180 women aged 19-42 who underwent embryo transfer and opted to use the aneuploidy screening test (iDAScore, MitoScore, and endometrial thickness registration were before the transfer).

**Results and Discussion:** iDAScore and biopsy day showed differences between implanted and non-implanted embryos. There was no difference between oocyte age, patient age, Gardner criteria, MitoScore, and endometrial thickness. However, while the dispersion of the MitoScore indicates a pattern towards low scores, implanted embryos have few high values compared to non-implanted embryos. The area under the curve is 0.63 with the iDAScore tool for the implementation assessment. This value varies depending on oocyte age, biopsy day, and Gardner criteria. The optimal cut-off point is 7.7, prioritizing sensitivity at 70% and expecting to obtain 50% false positives. Lowering the cut-off point would increase sensitivity and the number of false positives. Increasing the cut-off point reduces the number of false positives but increases the percentage of false negatives due to reduced sensitivity. There was an association between the iDAScore tool and embryo implantation. In the multivariate model, for each additional iDAScore point, the prevalence of implantation increases by 14%, adjusting for oocyte age and biopsy day. In addition, the prevalence of implantation in embryos biopsied on day 6 was 41% lower than in embryos biopsied on day 5.

**Conclusion:** Our results show that the predictive ability of the iDAScore can correctly classify implantation and non-implantation potential at a better rate than chance but does not discriminate effectively. Furthermore, performance is likely to vary according to oocyte age, biopsy day, and Gardner criteria. We suggest conducting studies with a larger sample size to assess the characteristics where the iDAScore performs better.

**P-04. Incidence of uterine polyps in women who undergo in vitro fertilization: Experience of an Assisted Reproduction Center**

**Emilio Blum-Panchana^1,2^, Annia Blum-Panchana^1,2^, Bernardo Blum-Pinto^1^, Medardo Blum-Narváez^1^, Xavier Blum-Rojas^1^, Tatiana Puga-Torres^1^**

^1^lnnaifest Assisted Reproduction Center, Guayaquil, Ecuador.

^2^Medical School, Universidad Espiritu Santo (UEES), Samborondon, Ecuador.

**Introduction:** Endometrial polyps can cause infertility and affect embryo implantation in patients undergoing in vitro fertilization, and it appears that location of the polyps may be one of the reasons for infertility. Endometrial polyps are the most described, while tubal and utero-tubal junction polyps are the least frequent.

**Materials and Methods:** A retrospective study was performed from 207 patients who underwent diagnostic hysteroscopy before starting in vitro fertilization treatment. Hysteroscopy was performed with a flexible hysteroscope under sedation. The results were evaluated by t student test with *p*<0.05 and result were expressed as mean ± standard error.

**Results:** Hysteroscopy revealed uterine pathologies in 54 patients. Endometrial polyps were the pathology with the highest incidence, while only one woman presented ostia polyps. Also women with endometrial polyps were older than women with cervical polyps or without pathologies (Table 1).

**Discussion:** The high incidence of endometrial polyps in comparison with other uterine pathologies, reaffirms our findings published previously, while this is the first time we report a case of ostia polyps in a patient. In this case, polyps present in both ostium may be the cause of metrorrhagia and infertility for five years. Even though ultrasound or sonohysterography allow us to visualize the uterine cavity, diagnostic hysteroscopy provides a clearer image of the endometrial polyps. This image technique is not yet considered routine before in vitro fertilization in assisted reproduction centers, our results support its use prevails over other diagnostic types to evaluate uterine cavity conditions.

**Conclusion:** Endometrial polyps are the uterine pathology with more incidence in patients with infertility who will undergo in vitro fertilization, therefore the study of uterine cavity by diagnostic imaging techniques such as hysteroscopy, ultrasound, or sonohysterography should be considered before starting *in vitro* fertilization.

**P-05. First experiences with non-invasive preimplantation genetic testing for aneuploidies: comparison of implantation and clinical pregnancy rate with elective FET**

**Adriana Gosálbez Ferrándiz^1^, Victor M Montes de Oca^1^, Randolfo Medina^2^, Juliana Martins^1^, Alicia Santos^1^**

^1^PROFERT, Programa de Fertilización Asistida y Medicina Perinatal. Santo Domingo, República Dominicana.

^2^Fertigenetics. Caracas, Venezuela.

**Introduction:** The existence of limitations with PGT-A such as the invasive nature of the biopsy procedure and the need for technical expertise is leading to increase use of non-invasive PGT-A (niPGT-A) using spent medium by embryos analyzed. A retrospective cohort study in a private center has been necessary to determinate the characteristics of patients, first results obtained and the clinical benefit of niPGT-A.

**Material and Methods:** Retrospective cohort study conducted from May 2022-March 2023. All IVF stimulation, luteal phase support protocols, and laboratory workflow were performed according to standard clinical practices, including specific niPGT-A protocols.

All embryos analyzed were inseminated with conventional IVF (82.6%) and ICSI (17.4%) and cultured to the blastocyst stage (day 6 or 7 post-insemination). Embryos were cultured in G-TL™ medium (Vitrolife). Spent culture medium (SMC) was collected on day 6-7, according to niPGT-A Igenomix protocol. Blastocyst were graded according to Gardner classification and divided in top quality: AA; high quality: A/B and poor quality A/C,B/C and CC. Blastocyst formed were vitrified using Cryotec method. Analyzed embryos have been divided into three groups according to women age, Group 1 (3 subjects ≤35 years), Group 2 (5 subjects: 36-38 years) and Group 3 (9 subjects ≥39 years). Implantation and ongoing pregnancy outcomes were compared (*p*-value) with frozen embryo transfer (FET) performed between 2021-2022 without niPGT-A.

**Results:** Average age of couples included in the study was 38.51±0,29, being the average age of women 38.22±3.9 and men 38.8±5.4. No significant differences were observed between groups regarding of blastocyst arrival rate. Main reasons why couples decided to undergo niPGT-A were women age (46.2%), male factor (23.1%) and RIF (15.4%). A total of 40 SCMs from 18 couples were collected. No paternal/maternal contamination was observed. Presence of Y chromosome was detected in 13 SMCs (32.5%) versus non-Y chromosome in 25 SMCs (62.5%); in two cases non-informative results were reported (5.0%). Most blastocysts obtained were classified as high quality (n=25: 62.5%), followed by poor quality (n=9: 22.5%) and top-quality blastocysts (n=6: 15.0%). Most frequent aneuploidies were in 16 chromosome (18.0%) followed by 18 and 20 chromosomes aneuploidies (8.0%, respectively). The comparison of results of niPGT-A group versus elective FET are described in Table 1.

**Discussion:** Advanced maternal age and male factor were the main causes of niPGT-A among the couples included, reporting an awareness of the influence of both sexes on embryonic development. Aneuploidies of 16 chromosome have been frequently observed; these are incompatible with life and end in pregnancy loss during the first trimester. No significant differences were observed between niPGT-A and elective FET groups in terms of implantation and clinical pregnancy rate.

**Conclusions:** Based on the results obtained, niPGT-A is a good alternative to assess the chromosomal status of embryos. When comparing two groups, no significant differences were obtained. In this study small number of samples is a limitation reason why large-scale randomized studies will be necessary.

**P-06. Successful Eye Movement Desensitization and Reprocessing (EMDR) technique for Vaginism to ease embryo transfer in *In Vitro* Fertilization**

**María Antonietta López^1^, Sophia Behrens^2^, Sadek Besereni^2^, María Teresa Urbina^2^, Randolfo Medina^2^, Luis Segura^2^**

^1^Psicotrauma, Valencia Venezuela.

^2^Fertigenetics, Caracas, Venezuela.

**Introduction:** Vaginismus is a sexual dysfunction, described as the spam of paravaginal muscles preventing sexual intercourse. A phobic attitude, pain, or the fear of pain may contribute to its persistence. Vaginismus impacts assisted reproductive techniques, troubling transvaginal ultrasound examination, intrauterine insemination, oocyte retrieval, and embryo transfer. Various approach techniques for vaginismus, have been proposed. Results of a Cochrane systematic review indicate that there is no statistically significant difference between systematic desensitization versus any of these strategies: group therapy plus systematic desensitization, *in vitro* desensitization, pelvic floor exercises, hypnotherapy, bibliotherapy, or waiting list. However, they found the evidence of the efficacy of treatments or interventions is limited. A novel approach for vaginismus is the psychotherapy of Eye Movement Desensitization and Reprocessing (EMDR). The clinical efficiency of EMDR in trauma-related disorders has been demonstrated in randomized trials and numerous meta-analyses and is recommended as the first-line treatment for post-traumatic stress disorder (PTSD) by the World Health Organization. As one of the causes of the vaginismus could be PTSD, EMDR therapy could be used. During EMDR sessions, patients are asked to attend simultaneously to the chosen target (e.g., the traumatic memory) and to an alternative tactile, auditory, or visual bilateral stimulus (BLS), which induces a REM-Sleep-like brain state. BLS may help process memories as REM reinforces memory consolidation, to facilitate reprocessing of maladaptive memories that are thought to be important to PTSD. Two cases of vaginismus were treated with EMDR (Torun *et al*., 2010), with successful results that persisted at two months follow-up. Herein we report one IVF patient that presented vaginismus, related to PTSD, treated with EMDR to ease embryo transfer in In vitro Fertilization.

**Material and Methods:** Female patient: 42 years old with endometriosis, bilateral sactosalpinx, and immunologic factor, married to an oligospermic man aged 44, three prior IVF-ET attempts with embryo difficult transfers in another fertility center.The mock embryo transfer was difficult because of vaginismus. Even under sedation, it was impossible to open the valves of the speculum. She underwent treatments such as using vaginal dilators, without good results. Then, she was referred to a psychologist.

Psychological treatment:

EMDR was used during 16 sessions, following an eight-phase protocol:

1. History and treatment plan

2. Introduction to EMDR protocol, and development of coping strategies

3. Evaluation of the treatment targets

4. Desensitization and reprocessing

5. Incorporation of positive cognitions

6. Body scan (and reprocess of any remaining bodily negative sensation)

7. Relaxation (re-establish emotional stability if distress has been experienced and for use between sessions)

8. Re-evaluation

During EMDR sessions, auditory bilateral stimulation side to side was provided by the therapist to induce eye movement. The psychologist decided the adequate moment to proceed to the following phase and when to deepen a certain phase.The patient was asked to take a deep breath and to report whatever came up: memories, thought or sensations. BLS sessions were repeated until the discomfort associated with the memory vanishes.

**Results and Discussion:** The patient substantially reduced her self-reported and clinician-rated anxiety, and dysfunctional beliefs regarding sexual intercourse, allowing a successful embryo transfer. The patient got pregnant and successfully had her baby.

**Conclusion:** These findings support the notion that EMDR could be an effective treatment for patients with vaginismus of traumatic etiology to ease embryo transfer in *In vitro* Fertilization.

**P-07. #Oncofertility in Latin America: still not trending**

**Márcia Mendonça Carneiro^1,2^**

^1^Departamento de Ginecologia e Obstetrícia - Faculdade de Medicina da UFMG, Brazil.

^2^Clínica ORIGEN Medicina Reprodutiva, Belo Horizonte, MG, Brazil.

**Introduction:** According to the REDLARA 2019 report, most assisted reproduction (ART) procedures are carried out in in Brazil (n=63), followed by Mexico (n=40) and Argentina (n=28). In 2019 A total of 7990 initiated cycles for fertility preservation were reported in 2019 representing a 19.5% increase but unfortunately only 5.7% were due to cancer-related factors. Oncofertility is a new interdisciplinary area in fertility care which addresses the issue of gonadotoxicity associated with oncological treatments and aims at preserving the reproductive potential. Despite being available for years, such fertility issues remain overlooked and at times inadequately addressed despite the availability of resources. Google is often used as a source of medical information by the lay population but unfortunately the quality of such information is poor and unreliable. Therefore knowing the needs of those who seek guidance online can help develop educational tools and improve care. Thus the objective was to investigate oncofertility care policies, guidelines, recommendations and services as well as the availability of oncofertility care in Argentina, Brazil and Mexico electronic search of public available data. We also aimed to identify search trends on Google using the terms "oncofertility", "cancer and pregnancy", “breast cancer and pregnancy”, "fertility preservation” and "fertility preservation and cancer”.

**Material and Methods:** A search was performed on Google Trends using the terms described above in the last 5 years in each country. Total results were collected, categorized and connected to a topic. Personal information was removed as well as data searched by few people, searches for a term performed by the same person in a short period of time and special characters. For each country Google trends calculated the percentage each term was searched. Pages containing information related to oncofertility to the public were eligible. As individuals rarely examine more than the first 3 pages of a search, information obtained from the first 3 pages obtained in the Google search were evaluated. Pages identified were classified as scientific, lay media, healthcare professional and other. No ethical approval was necessary as only public available online information was used.

**Results:** Google trends did not show any data in the three countries for the terms searched as they apparently are rarely included in searches. Oncofertility and fertility preservation are rarely searched in Google while fertility preservation and cancer is not searched at all. Cancer and pregnancy was searched in Brazil but numbers were too low to be compiled. Google search revealed 10 links for websites in each page in Brazil and Argentina while in Mexico only 2 pages were identified. The contents classification in these pages were: 15 scientific in Argentina, 4 in Brazil and 4 in Mexico. Lay media content was available in 9 Argentinian, 8 Mexican and 6 Brazilian pages. Healthcare professionals were responsible for content in 2 Mexican , 3 Argentinian, and 16 Brazilian websites. National oncofertility care guidelines were available only in Argentina.

**Conclusion:** Oncofertility and its related terms do not raise the interest of people shown by the lack of trending in Google in Argentina, Brazil and Mexico. The results shown here reflect the low usage in fertility preservation for cancer in Latin America. The development of online patient education resources on oncofertility and fertility preservation may be important tools to educate people and improve oncologic and fertility care as Google is the most popular search engine and people often use it to obtain medical information. Moreover, available information on the topics is scarce and heterogeneous. As the burden of cancer is expected to grow in Latin America, there is a need to inform and educate about oncofertility.

**P-08. Deferred embryo transfer, following a freeze-all strategy, improves clinical pregnancy rate in patients with few available embryos**

**S Romero^1^, R Pella^1^, F Escudero1, K Pérez^1^, M García^1^, P Orihuela^1^**

^1^Centro de Fertilidad y Reproducción Asistida CEFRA S.A.C., Lima, Peru.

**Introduction:** In recent years, with the aim of improving the outcomes of embryo transfers, relevance has been given to the understanding of the implantation window, however oocyte and embryo quality are key factors that are not to be disregarded. Although a freeze-all strategy and subsequent frozen embryo transfer(s) has been suggested as a way to improve pregnancy rates; it is unclear whether this strategy benefits all kind of patients (i.e. with or without surplus embryos, etc). In this study, we aim to provide an answer on which patients may benefit of a freeze-all policy and a subsequent frozen embryo transfer.

**Material and Methods:** This retrospective cohort study includes infertile patients aged 21 to 44 years old, without previous history of recurrent failure of ART (including recurrent miscarriages). Enrolments took place between January 2015 and November 2021 and cycles with oocyte donation and PGT were excluded. Blastocyst transfers were performed in: 1) a fresh cycle (ET) or 2) a deferred cycle with surplus frozen embryos (FET) or embryos that were frozen in a freeze-all policy (FET-FA).

The number of cycles complying with the inclusion criteria were 662 ICSI cycles. Fresh embryo transfers (ET) were performed in 377 cycles (59 with a subsequent Frozen embryo transfer, FET). Frozen embryo transfers (without prior ET) were performed in 285 cycles.

**Results:** Following the first transfer (either ET and FET-FA), CPR was 40.8% and 55.8% (ET and FET-FA, respectively). Following the subset analysis of 2 age groups (≤38 & >38 years-old); in the ≤38-group, CPR was 45.5% and 56.2% (ET and FET-FA, respectively), while in >38-group, the rates were 30.8% and 52.8% (ET and FET-FA, respectively); *p*<0.05. CCPR was also significantly better in the FET-FA group: 52.1% *vs*. 62.7% and 34.2% *vs*. 58.3% in the ≤38-group and >38-group, respectively. Additionally, CPR was analyzed for patients with ≤2 usable embryos (1 attempt) or ≥3 usable embryos (surplus embryos after first attempt). When a single attempt was possible, CPR was 31.4% and 58.1% (ET and FET-FA, respectively); *p*<0.05. However, when surplus embryos were available, no difference in CPR (or CCPR) between ET and FET-FA groups were observed.

**Conclusion:** In our setting, the data suggests that a freeze-all strategy (with subsequent frozen embryo transfer) over fresh transfer is advantageous for patients with few available embryos (1 or 2 embryos for a single attempt), increasing the chances to pregnancy by 85%.

**P-10. Intraovarian PRP injection to improve the number of euploid embryos obtained during *in vitro* fertilization**

**Moisés Sánchez^1^, Tania Castro^1^, José Gonzales^1^, Jonathan Vasquez^1^**

^1^Unidad de Medicina Reproductiva, Centro de Fertilidad GERMINAR, Lima, Peru.

**Introduction:** Ovarian aging is a physiological process that leads to a decline in oocytes quantity and quality, negatively impacting the formation of euploid embryos during in vitro fertilization treatment. Platelet-rich plasma (PRP) is rich in growth factors and cytokines which play key roles in tissue repair and regeneration. In this context, PRP treatment has recently been used as an adjunct in assisted re¬production technology, specially, as an intraovarian injection in conjunction with IVF for women who have poor ovarian reserve, pre¬mature ovarian insufficiency, and even menopause, showing improvement in markers of ovarian reserve. The goal of this work was to evaluate if the intraovarian injection of PRP increases the number of euploid embryos achived in patients who failed to obtain any euploid embryo in a cycle prior to PRP.

**Material and Methods:** 29 patients underwent 2 stimulation cycles for in vitro fertilization (Table 1). In the first cycle, after follicular aspiration, homologous PRP was injected into the ovaries meanwhile the oocytes were vitrified. After the second follicular stimulation and aspiration, all oocytes, both fresh and vitrified, underwent in vitro fertilization. Embryos were cultured and subjected to embryo biopsy on the fifth or sixth day of development. The biopsies were analyzed by PGT-A. For statistical analysis the proportion of euploid and non-euploid embryos in the pre and post PRP cycle was analyzed with the test of equality of proportions. In addition, the proportion of patients who managed to have at least one euploid embryo compared to the patients who did not obtain any in the first cycle was analyzed with the exact binomial test.

**Results:** No significant difference was found in the proportion of euploid embryos obtained from both pre (38%) and post (45%) PRP cycle (*p*=0.589). However, from all the data, of the 18 patients who failed to obtain any euploid embryo in the pre-PRP cycle, 8 managed to obtain at least 1 euploid embryo post-PRP, showing that a patient without positive results in the first cycle can increase its probability of obtain at least 1 euploid embryo to 0.44 with a 95% CI of (0.22 - 0.69).

**Discussion:** Previous works have shown the beneficial effect of intra-ovarian PRP administration in improving ovarian reserve markers, such as serum an¬ti-Müllerian hormone, and in¬creased oocyte and embryo retrieval. Likewise, in the present work an increase in the rate of euploid embryos was found, results that are in agreement with previous works. Additionally, it was found that from the group of patients who failed to obtain any euploid embryo in the cycle prior to PRP, it was possible to obtain at least one euploid embryo after PRP administration, very encouraging results which show that PRP would represent an alternative to achieve a pregnancy with their own eggs for those patients who would otherwise have to appeal to egg donation.

**Conclusion:** Intraovarian PRP administration represents an alternative for increase the number of euploid embryos achived through *in vitro* fertilization, especially in patients who were unable to obtain any euploid embryo prior to the administration of PRP.

**P-09. GnRH agonist trigger improves the yield of usable Blastocysts**

**S. Romero^1^, R. Pella^1^, F. Escudero^1^, K. Pérez^1^, M. García^1^, P. Orihuela^1^**

^1^Centro de Fertilidad y Reproducción Asistida CEFRA S.A.C., Lima, Peru.

**Introduction:** It is well documented that oocyte and embryo quality worsens with the aging of the patient, and this has an impact on the yield of usable blastocysts that are obtained per initiated cycle. However, no differences are expected in terms of the yield of MII oocytes at oocyte retrieval. Because the ovarian reserve (by dosing AntiMüllerian hormone) is considered a reference parameter for the expected number of oocytes at retrieval. We evaluated whether different triggers would have an impact on the yield of oocytes retrieved as well as the yield of usable Blastocysts.

**Material and Methods:** This retrospective cohort study includes infertile patients aged 26 to 46 years old, without previous history of recurrent failure of ART (including recurrent miscarriages). Enrolments took place between August 2017 and December 2022. Cycles with oocyte donation were excluded. Patients received a personalized stimulation protocol, but as a general rule, patients started with a standard short antagonist protocol. Ovulation was triggered by hCG, GnRH agonist or a combination of both (Mix). The number of cycles complying with the inclusion criteria were: 2060 (1421 in which oocytes were fertilized by ICSI, 595 in which all oocytes were vitrified, 16 in which part of the oocytes were vitrified and part fertilized by ICSI, and 28 in which no MII oocytes were obtained). AMH values dating no longer than 12 months prior to the IVF cycle were included in the analysis (n=607).

**Results:** Frequency distribution for AMH showed that median AMH values was 1.2. Patients with better response to ovarian stimulation were triggered for ovulation with GnRH agonist (14.0±7.7 retrieved oocytes). While hCG and Mix had 7.4±5.0 and 8.2±5.4, retrieved oocytes, respectively. Similarly, GnRH agonist was used in patients with an AMH value of 2.6±1.9; while hCG or Mix were used when AMH values were 1.1±1.0 and 1.4±1.5, respectively. The linear regression for AMH versus the number of MII oocytes, showed that the slopes of hCG vs GnRH agonist were not different (1.59 and 1.49), while slope of Mix was significantly lower (0.81) than hCG and GnRH agonist (*p*<0.05). Moreover, the Y-intercept was higher for GnRH agonist. In contrast, the linear regression for AMH versus the number of usable blastocysts (either frozen or transferred) showed that the slopes of the three triggers did not differ significantly. However, the Y-intercept was again higher for the GnRH agonist. In both cases, AMH and the number of MII or usable blastocysts were positively correlated. All together this suggest that independently of the AMH value, the GnRH agonist yields more MII oocytes and usable blastocysts.

**Conclusion:** In our setting, the data suggests the GnRH agonist trigger improves the yield of MII oocytes and usable blastocysts.

**P-11. Relationship between male obesity and sperm DNA fragmentation**

**Lizeth Padilla^1,2,3^, Tania Castro^1,2,3^, Santiago Díaz^2^, Santiago Llatas^3^, Cristian Perez^3^, Flor Carvallo^1^, José Gonzales^1,2,3^, Jonathan Vásquez^1,2,3^**

^1^Unidad de Medicina Reproductiva, Centro de Fertilidad GERMINAR, Lima, Peru.

^2^Unidad de Medicina Reproductiva, Centro de Fertilidad de Cajamarca, Cajamarca, Peru.

^3^Unidad de Medicina Reproductiva, Clínica de Fertilidad del Norte CLINIFER, Chiclayo, Peru.

**Introduction:** Obesity is a widely recognized risk factor for female fertility. During the last decades, in global population has been suggested a relationship between the increase in male obesity and the decrease in semen quality, mainly affecting semen parameters such as sperm count. In addition, it is increasingly being recognized that seminogram analysis has limited utility in the evaluation of male infertility, which is why evaluation with complementary tests such as sperm DNA fragmentation is currently suggested for a better evaluation of seminal quality. The aim of this study was to determine whether body mass index (BMI) affects the proportion of men with abnormal sperm DNA fragmentation index (DFI) in a population of patients who attended an infertility clinic.

**Material and Methods:** 850 seminal samples collected between 2021 - 2022 in 3 fertility centers in Peru were analyzed. Patients with more than 5 days of sexual abstinence, azoospermic and cryptozoospermic were excluded. The seminal samples were collected by masturbation. Patients weight and height were recorded the same day to get the BMI. The evaluation of sperm DNA fragmentation was performed using the Sperm Chromatin Dispersion (SCD) technique using the commercial kit CANFrag (*Candore, India*). All samples were previously subjected to an REM test using the density gradient technique or centrifugal washing. The %DFI was determined by counting 200 spermatozoa, considering those greater than 17% as altered. Patients were assigned to 3 different groups according to their BMI: normal weight (BMI: 18.5-24.9 kg/m^2^), overweight (25.0-29.9 kg/m^2^), or obese (≥30.0 kg/m^2^). Additionally, patients were divided into 3 age groups: 18 to 30 years, 31 to 40 years, and 41 years and over. Statistical analysis of the data was performed using the chi-square test of independence.

**Results:** No relationship between BMI and the proportion of patients with altered % DFI was found in any of the 3 age groups studied (Table 1).

**Discussion:** At present it is not yet clear whether obesity plays a negative role in the DNA fragmentation rate. Mir *et al* in 2017 and Fariello *et al*. in 2012, for example, found a correlation between the DFI and BMI of the patients studied, identifying that people with a higher obesity index not only had a higher level of fragmentation but also presented decreased fertility parameters. Meanwhile, Dupont *et al*. in 2013 found significant differences only in patients with obesity, but not in overweight patients. On the other hand, Sepidarkish *et al.* in 2020 in their meta-analysis with 8255 patients could not demonstrate a direct correlation between obesity and %DFI. This wide range of results may be due to factors such as the different techniques used for the DFI evaluation, different threshold to determine normality and the different populations studied, among others. In addition, should be take into account that, unlike the previous works, we performing a REM test prior to the DNA fragmentation technique which is more clinical useful.

**Conclusion:** Male obesity represented by the BMI value does not alter the proportion of patients with altered %DFI regardless of the age of the man under study.

**P-12. Male age and its relationship with sperm DNA fragmentation**

**Jazmin Villalobos^1,2,3^, Santiago Díaz^2^, Santiago Llatas^3^, Cristian Pérez^3^, Flor Carvallo^1^, José Gonzales^1,2,3^, Jonathan Vasquez^1,2,3^**

^1^Unidad de Medicina Reproductiva, Centro de Fertilidad GERMINAR, Lima, Peru.

^2^Unidad de Medicina Reproductiva, Centro de Fertilidad de Cajamarca, Cajamarca, Peru.

^3^Unidad de Medicina Reproductiva, Clínica de Fertilidad del Norte CLINIFER, Chiclayo, Peru.

**Introduction:** Increasing male age has been related to the appearance of multiple dysfunctions that have a significant impact in sperm quality, such as altered testicular function and hypothalamic-pituitary axis, decreased antioxidant capacity, among others. On the other hand, damage to sperm DNA, measured through sperm DNA fragmentation index (DFI) seems to be related to increasing age. Our objectives were to determine if male age is directly related to the DFI and to indicate a specific age from which a noticeable increase in DFI occurs. **Materials and Methods:** We analyzed 1512 semen samples collected during the years 2021 - 2022 in 3 fertility centers located in Peru. Azoospermic and cryptozoospermic patients were excluded. Regarding the statistical analysis, first the limit of days of abstinence (DA) was determined from which they could have a negative influence on the proportion of patients with altered DFI (DFI>17%). Subsequently, data analysis was performed only with patients who had DA less than or equal to the limited estabished before. Then we proceeded to determine the effect of age according to 4 age groups (<30, 31-40, 41-50, >50) on the proportion of patients with altered DFI using de Chi-square test of independence followed by logistic regression with their respective odds ratio. Finally, using the Davies test, the age at which the DFI drastically increases was determined. All analyses were performed with R 4.2.2 software and the "segmented" library was used to estimate the Davies test.

**Results:** It was determined that the proportion of patients with altered DFI increases when DA are>6, while from the age of 45 years onwards it was evidenced as a break point for a significant increase in the proportion of patients with altered DFI (Figure 1). In addition, a direct relation between increasing age and altered DFI was identified (Table 1). Likewise, significant differences (p<0.05) were found in the IDF value, which were 1.81, 2.77 and 4.40 times higher than the reference (under 30 years) for the age ranges 31-40, 41-50 and >50, respectively (Figure 2).

**Discussion:** Different studies show a clear association between advancing paternal age and the DFI of an individual, given the overproduction of reactive oxygen species and the increasing number of mutations occurring in the germ cell line. In the present work an increase of patients with altered DFI after 45 years of age is observed, in agreement with previous studies, which indicate that increasing age leads to defective chromatin packaging and apoptotic disorders, which finally generate gametes with defective DNA.

**Conclusion:** It is concluded that age is indeed a relevant factor influencing the proportion of patients with normal DFI, being quite significant after 45 years of age.

**P-13. Is the spermogram a sufficient evaluation in normozoospermic patients?**

**Jazmin Villalobos^1,2,3^, Santiago Díaz^2^, Santiago Llatas^3^, Cristian Pérez^3^, Flor Carvallo^1^, José Gonzales^1,2,3^, Jonathan Vásquez^1,2,3^**

^1^Unidad De Medicina Reproductiva, Centro de Fertilidad y Reproducción Asistida GERMINAR, Lima, Peru.

^2^Unidad De Medicina Reproductiva, Centro de Fertilidad de Cajamarca, Cajamarca, Peru.

^3^Unidad De Medicina Reproductiva, Centro de Fertilidad del Norte CLINIFER, Chiclayo, Peru.

**Introduction:** Traditionally, the spermogram has been considered the only exploratory test to estimate male fertility. However, in the last decade the evaluation of sperm DNA fragmentation (SDF) has been postulated as an important complementary test for a more comprehensive assessment of spermatozoa. Currently, this test is required by many fertility centers prior to a highly complex assisted reproduction treatment. However, there is reluctance to prescribe this test for various reasons, even when there is a previous spermogram with a result of normozoospermia. Therefore, the aim of this study is to evaluate the proportion of patients with altered sperm DNA fragmentation index (DFI) in normozoospermic patients and by age range.

**Materials and Methods:** 1663 semen samples were analyzed during the years 2021 - 2022 in 3 fertility centers located in Peru. The 1083 semen samples with a diagnosis of normozoospermia were selected for this study. Evaluation of sperm DNA fragmentation was performed using the Sperm Chromatin Dispersion (SCD) technique using the commercial CANFrag kit (Candore, India), and all samples were previously subjected to sperm recovery using the density gradient technique. The DFI was determined by counting 200 spermatozoa, considering as an altered value those higher than 17%. Regarding statistical analysis, the proportion of patients with normal and altered DFI was evaluated by logistic regression with their respective odds ratios. All analyses were performed with R 4.2.2 software.

Results: It was found that 174 patients of the 1083 evaluated had an altered DFI value (16.07%). In addition, the proportion of patients with altered DFI was 9%, 14%, 20%, 29% in the age ranges of <30, 31-40, 41-50 and >50 respectively, observing a direct relationship between age and DFI (Table 1). Furthermore, the risk of having altered DFI was 1.70, 2.67 and 4.30 times higher than the referent (younger than 30 years) for age ranges 31-40, 41-50 and >50, respectively, and being significantly increased from the group aged 41 years and older (Figure 1).

**Discussion:** Several studies have analyzed the behavior of DFI in normozoospermic groups, finding that there is not always a direct relationship between spermogram and DFI results. In the present study is observed that even in the younger age groups there is a proportion of patients with altered DFI, which increases as age increases. Different mechanisms have been postulated which may increase sperm DNA fragmentation without impairing semen values, such as oxidative stress. Using spermatozoa with damaged DNA in assisted reproductive techniques can result in low fertilization rates, poor embryo development, lower pregnancy rates or high miscarriage rates. For this reason, it is considered necessary to establish the IDF of patients even diagnosed as normozoospermic and regardless of age before assisted reproduction treatment, in order to prevent insemination of eggs with sperm that have damaged genetic material. Likewise, it is suggested to propose to the patient some type of sperm selection that helps us to considerably reduce the amount of sperm with fragmented DNA.

**Conclusions:** Patients with normozoospermia can also present altered DFI, this risk being increased in men older than 40 years.

**P-15. Affectation of seminal parameters in HIV-seropositive patients undergoing seminal washed**

**Pedro Cuapio Padilla^1^, Mirna Guadalupe Echavarría Sánchez^2^, Carlos Gerardo Salazar López Ortiz^3^**

^1^Laboratorio de Andrología, Banco de Semen, Calidad e Investigación. Hisparep, Clínica de Reproducción Asistida, Hospital Español. Mexico City, Mexico.

^2^Andrología Clínica. Hisparep, Clínica de Reproducción Asistida, Hospital Español Mexico City, Mexico.

^3^Biología de la Reproducción. Hisparep, Clínica de Reproducción Asistida, Hospital Español, Mexico City, Mexico.

**Introduction:** Currently worldwide, the HIV virus (human immunodeficiency virus) affects millions of men worldwide, who currently want to have offspring and whose age range is approximately 20 to 50 years. The use of retrovirals has allowed the disease to be controlled and the use of condoms has allowed the respective couples to reduce the infection, but prevents pregnancy. Currently, the semen washing technique for infectious samples such as HIV is available in some clinics worldwide. This technique makes it possible to eliminate seminal fluid, decrease viral load and select good quality sperm. Subsequently, the HIV semen wash must be frozen and a part sent to carry out the confirmatory test by real-time PCR.

**Material and Methods:** HIV semen washing was performed on 132 seropositive patients with different and undetectable viral loads, prior review of the necessary studies and informed consent. The age range between 21 and 62 years.

**Results:** The results show that the seminal quality of the patient samples is affected by the presence of the virus, affecting the morphology (defects in the head, middle part, tail and cytoplasmic residues). Therefore, an increase in the rates of teratozoospermia, deformity and multiple abnormalities is seen. The etiologies that were presented in the 132 seminal samples, 11 samples (8.3%) presented Normozoospermia, 105 (79.5%) Teratozoospermia, 3 (2.3%), 1 (0.8%) Asthenoteratonecrozoospermia, 9 (6.85%) Asthenoteratozoospermia, 3 (2.3%) Oligoastenoteratonecrozoospermia and 3 (2.3%) Oligoteratozoospermia. The average of the progressive mobility in fresh 44.95±8.34 and post thawing 27.8±11.6. In fresh samples, the average for morphology (%) is 2.35±0.81. The real-time PCR molecular result showed 128 HIV Negative semen washes (96.97%), 3 Positives (2.27%) and 1 non-amplified (0.76%). The post-thaw test shows that there is a sufficient concentration, mobility and viability to carry out in vitro (ICSI) cycles.

**Conclusion:** The results show that the adequate evaluation of the samples is being carried out, taking into account the sperm quality, use of the facilities, technology and methodology for washing, as well as the subsequent freezing and that the real-time PCR allows the detection of the presence of virus in washed HIV samples.

**P-14. How old is a semen analysis recorded useful for an IVF cycle?**

**Natalibeth Barrera^1,2^, Carla Bonelli^1,2^, Jimena Alciaturi^1,2^, Carolina Surka^1^, Andrea Torrens^1^, Rosina Ordoqui^1^, Lidia Cantú^1,2^**

^1^Reprovita, Laboratorio de Andrología, Montevideo, Uruguay.

^2^Centro Esterilidad Montevideo (CEM), Montevideo, Uruguay.

**Introduction:** Study Question: Is the semen analysis prior to an IVF cycle predictive of the sample collected on the day of the procedure?

**Materials and Methods:** Study design: a retrospective descriptive study of 99 semen samples used in IVF/ICSI cycles between January-June, 2022 was performed. The variables semen analysis date, total sperm count, and percentage of progressive motility (PM) were collected from the semen analysis history. The results obtained were compared with the parameters observed on the day of the procedure. The samples were divided into groups according to the date of the antecedent: Group 1, Sperm Test ≤ 210 days old and group 2 more than 210 days old. Statistical analysis was performed using the XLSTAT software (Addinsoft, USA). A *p* value <0.05 was considered significant.

**Results:** 63% of the patients studied had a sperm analysis prior to their IVF/ICSI cycles older than 365 days old (x̅=430.61 days). Significant differences were observed between groups 1 and 2. Group 1, the count of the sample provided on the day of the procedure differed by less than 18.52% with respect to the total count of the sample provided on their last sperm analysis recorded, while in Group 2, the differences declined by minus 87.60%. No differences were observed in PM.

**Discussion:** Limitations of the study: future studies should include other sperm variables, such as percentage of fragmented spermatozoa and morphology.

**Conclusion:** Implications of the findings: this study allowed us to determine that a semen analysis > 210 days is not predictive of the total sperm count on the day of the procedure. Therefore, physicians should advise their patients to have a semen analysis close to the aspiration date, which would have more predictive value and would help the laboratory staff in better prepare male history background for the day of the procedure. Letting embryologist to select semen sample preparation method according to history, insemination technique and finally having an impact on the cycle outcome. The patient should be advised to have a semen analysis in the 6 months prior to the follicular aspiration date.

**P-17. Is embryo morphology still relevant in euploid embryos for implantation rates?**

**Gigliana Catanzaro Foppiano^1^, Eduardo Gazzo Benavides^1^, Fernando Pena Espinoza^1^, Rocio Davalos Torres^1^, Jose Luis Llanos Carrillo^1^, Francesco Foppiano Florez Estrada^2^, Mario Ascenzo Battistini^1^, Marcelo Velit Suarez^1^, Luis Ernesto Escudero Velando^1^**

^1^Inmater Fertility Clinic, Lima, Peru.

^2^Pediatric Allergology Department of Pediatrics, Dr von Hauner Children's Hospital, Munich, Germany.

**Introduction:** The scoring system for human blastocysts is traditionally based on morphology but, there are controversies on the effect of morphology parameters on implantation rates. Advances in genetics, culture media and vitrification techniques, allow us to select euploid blastocyst that can be used in frozen-thawed embryo transfer (FET). However, are there any additional parameters that help us choose the best euploid blastocyst to transfer? Objective: The aim of this study is to evaluate the association of the trophectoderm (TE) and the inner cell mass (ICM) with the implantation rate (IR) to establish which factor is more determinant in a setting of single euploid embryo frozen transfer.

**Material and Methods:** This study follows a retrospective cohort study design and uses clinical data form our fertility clinic. Data was collected between January 2018 and January 2022 and included 654 patients with no previous embryo transfers who received a single euploid frozen embryo. Patients with intrauterine conditions affecting pregnancy outcomes of FET were excluded. Euploid confirmation was made with PGT-A assessment (Igenomix^®^). The endometrium was prepared using standard hormone replacement therapy before embryo transfer. Blastocyst grading criteria are based on Gardner blastocyst grading system. Implantation rates were estimated and compared between different scores ”a“, “b“ and “c“ for the ICM and the TE independently using χ2 test. A composite score was created which considered both factors together; due to small numbers of embryos with “c” scores all embryos with a “c” score in ICM or TE were considered in one class denoted as “*c”. Their implantation rates were also compared using χ2 test. In addition, the association of the independent and the composite scores was assessed using logistic linear regression adjusting for potential confounders.

**Results and Discussion:** The overall implantation rate was 62.8% (CI95% [59.1%,66.5%]). The IR for ICM scores “a”, “b” and “c” were 67.5%, 55.1% and 26.7% respectively and significantly different (p=0.0005). The IR for TE scores “a”, “b” and “c” were 67.0%, 58.6% and 42.9%, respectively, and significantly different (p=0.009). This results show that independently, both factors have an effect on the outcome. The IR for the composite scores (ICM/TE) “aa”, “ab”, “ba”, “bb” and “*c” were 67.6%, 66.7%, 62.9%, 54.9% and 37.1% and were significantly different (p=0.0015). To assess which of the variables better explained the outcome, logistic regression models were fitted unadjusted and adjusted for oocyte age. The OR for the scores “ab”, “ba”, “bb” and “*c” compared to “aa” were 0.95 [0.60,1.56], 0.82 [0.40,1.71], 0.59 [0.39,0.88] and 0.29 [0.14,0.58], respectively. There was no significant difference in the IR between scores “aa”, “ab” and “ba”, however the OR of “ba” compared to “aa” was smaller than that of “ab”. Lack of significance might be because “ba” was the smallest group (35). The odds of implantation for the “bb” score was 41% less than “aa”. In addition, based on the Akaike Information Criterion (AIC) the adjusted model for ICM only score had the best goodness of fit.

**Conclusion:** We found no significant differences in the IR of embryos with a composite score (ICM/TE) “aa“, “ab“ or “ba“, whereas that of embryos with “bb“ was significantly lower. However, our data suggests based on the observed lower IR of “ba“ compared to “ab“ and the AIC of our regression models that the ICM score is more determinant.

**P-18. The effect of endometrial thickness on implantation rate of single euploid embryo transfer cycles**

**Gigliana Catanzaro Foppiano^1^, Eduardo Gazzo Benavides^1^, Fernando Pena Espinoza^1^, Rocio Davalos Torres^1^, Jose Luis Llanos Carrillo^1^, Francesco Foppiano Florez Estrada^2^, Mario Ascenzo Battistini^1^, Marcelo Velit Suarez^1^, Luis Ernesto Escudero Velando^1^**

^1^Inmater Fertility Clinic, Lima, Peru.

^2^Pediatric Allergology Department of Pediatrics, Dr von Hauner Children's Hospital, Munich, Germany.

**Introduction:** The relationship between endometrial thickness and implantation rate remains poorly defined and evidence is contradicting. On the other hand, embryo quality improves significantly pregnancy outcomes. Recent advances in genomic technology have revolutionized the field of assisted reproduction, making it possible for example to establish the euploid status of embryos. Few studies have investigated the effect of transferring only euploid embryos on the relationship between endometrial thickness and implantation rate, therefore our objective was to assess the association between the endometrial thickness and the implantation rate when only euploid frozen embryos are transferred.

**Material and Methods:** This study follows a retrospective cohort study design and uses clinical data form our fertility clinic. Data was collected between January 2018 and January 2022 and included 654 patients with no previous embryo transfers who received a single euploid frozen embryo. Patients with intrauterine conditions affecting pregnancy outcomes of FET were excluded. Euploid confirmation was made with PGT-A assessment (Igenomix^®^). The endometrium was prepared using standard hormone replacement therapy and endometrial thickness was measured before embryo transfer using transvaginal ultrasound in millimeters. Comparisons of the implantation rates between different ranges of endometrial thickness was performed using *x*2 test. The association of endometrial thickness with the implantation rate was assessed using logistic regression and generalized additive models (GAM) were used to investigate non-linear associations.

**Results and Discussion:** The overall implantation rate in this cohort was 62.8% (CI95% [59.1%,66.5%]). We categorized patients based on the thickness of their endometrium (5 to 7mm, 8 to 9 mm, 10 to 11 mm, 12 to 16 mm) and compared their implantation rates, finding no significant difference (*p*=0.79). After performing logistic regression adjusting for the Oocyte age and recipient age we found no significant effect of endometrial thickness on the implantation rate (OR=1.02, CI95% [0.94,1.11]). Modeling the relationship of the endometrial thickness assuming a quadratic relationship using GAMs did not improved the model based on the Akaike information Criterion (AIC).

**Conclusion:** In this study, we found that an endometrial thickness between 5 and 16 mm has no significant effect on the implantation rate of euploid frozen embryo transfers.

**P-19. Our daily helplessness: its presence and experience in the outpatient operating room of the Assisted Reproduction clinic**

**Marcia Christina Gonçalves Gusmão^1^, Roberto de Azevedo Antunes^2^, Marcelo Marinho de Souza^2^, Ana Cristina Allemand Mancebo^3^, Bruna Stumpo Vaz^4^, Flavio Faria de Freitas^5^, Maria do Carmo Borges de Souza^2^**

^1^Psychoanalyst, Psychologist. Fertipraxis Centro de Reproducao Humana, Rio de Janeiro, Brazil.

^2^Clinicians, Clinic Directors. Fertipraxis Centro de Reproducao Humana, Rio de Janeiro, Brazil.

^3^Chief Embryologist. Fertipraxis Centro de Reproducao Humana, Rio de Janeiro, Brazil.

^4^Embryologist. Fertipraxis Centro de Reproducao Humana, Rio de Janeiro, Brazil.

^5^Pharmacist. Fertipraxis Centro de Reproducao Humana, Rio de Janeiro, Brazil.

**Introduction:** From the psychological interventions performed by the psychoanalyst in the Outpatient Surgical Center of a private assisted reproduction clinic it was possible to detect the presence of the feeling of helplessness, expressed in the patients' statements. Among many other feelings that emerge in patients before, during or after the procedures performed, we observed that the feeling of helplessness might not always be identified, supported or heard. Psychoanalytic theory was the basis for identifying and detecting helplessness. This study seeks to point out the need to identify the feeling of helplessness present in the frailties and vulnerabilities of patients who undergo assisted reproduction procedures in the outpatient operating room environment.

**Material and Methods:** A prospective study of care and psychological interventions performed in the outpatient surgical center (OSC) of the assisted reproduction clinic from January 2019 to December 2022. Patients are first attended by the nursing staff. They check vital signs, confirm the medications and previous laboratory tests performed. After an anamnesis with the anesthesiologist and the attending physician, the psychoanalyst presents herself and asks consent for listening/speaking, before, during and after the procedure. All data collected are recorded in the electronic medical record and shared with the team directly, when necessary.

**Results:** 1011 interviews were performed by the psychoanalyst, which correspond to 47% of 2149 OSC procedures performed in the clinic during the study period. At the time of oocyte pick-up for IVF/ICSI, the psychoanalyst was present in 595 cases (60%) of 1000 visits; in the 396 oocyte cryopreservation, 110 were interviewed (28%). Embryo transfers had psychological assistance in 306 cases, 41% of the total of 753 transfers. Patients observations were written in their medical records. Relevant points were shared and discussed with the medical and nursing staff directly.

**Conclusions:** Considering psychoanalytic theory as a guide for understanding the notion of helplessness, it was possible to identify, from listening to the patients' statements and the intersubjective relationships established with the clinical staff, the presence of this feeling in the OSC environment of an ART clinic. In view of helplessness as a structuring condition of the human psyche, identifying its presence and understanding its effects on the subjectivities of patients involved in ART procedures is necessary. Patients speech addressed to the psychoanalyst or to the multidisciplinary team in this environment contains the utterance of their feelings, conscious and unconscious, that affect their psyche. In this context, the feeling of helplessness, expressed and enunciated in the statements and conducts of patients as well as the team, may go unnoticed and not receive the necessary care. In the OSC environment, patients will often encounter their frailties, their faults, their psychic realities and the effects on the uniqueness of their stories. As a result of this experience, they are confronted with the reality that they would so much like to avoid, that is, to use the AR technique to achieve an unconsummated desire - pregnancy. The attention directed to helplessness can produce a greater understanding of its effect on the subjects, relationships and destiny that each will try to give to his existential helplessness. Faced with the difficulty of becoming pregnant, the helplessness of the "I", the AR technique can be a way. Psychoanalytic action can reflect and promote the understanding of "our daily helplessness", being able to welcome with their listening the subjects involved, whether they are patients or even the team.

**P-20. Men's psychological well-being, male gamete receptors in heterosexual couples**

**María Laura Cardozo Cal^1^**

^1^Master in Psychology of assisted human reproduction.

**Introduction:** Gamete Reception Assisted Reproduction is a treatment that provides an answer to couples or people who feel reproductive desire at some point in their lives and could not process their child on their own. The feeling of genetic disengagement is one of the most thoughtful reasons and that merits a deep reflection by those who are gamete recipients, when making decisions, to access or not to tradition with donation of gametes. In the case of reception of sperm as a maternity and paternity option the bereavement imbalances the couple, can produce a strong emotional impact on these patients, so it is necessary to assess whether the emotional state of the couples is altered to pass the genetic bereavement, appreciate the degree of decision-making for access to treatments, verify that there is agreement between both partners, accompanying these processes by psychological intervention. The present work will aim to think about the perception of well-being or psychological discomfort, understand the relative impact of the psychological characteristics of infertility generated in men members of heterosexual couples to undergo Assisted Reproduction Treatments of sperm reception, since it implies them to renounce to perform such treatments, with their own gametes, delving into the pain of genetic and identity loss; visualizing the coping styles of the male, in the face of emotional tension, generated by the fear of the unknown, the future bond with his son, the disappointment of not having the child imagined, which could be important contents for psychological interventions.

**P-22. Oocyte In Vitro Maturation, following oocyte “Capacitation” (CAPA-IVM) poses an alternative technique for ART patients**

**S. Romero^1^, R. Pella^1^, F. Escudero^1^, K. Pérez^1^, M. García^1^, P. Orihuela^1^**

^1^Centro de Fertilidad y Reproducción Asistida CEFRA S.A.C., Lima, Peru.

**Introduction:** Oocyte *In Vitro* maturation (IVM) refers to the maturation in vitro of immature COCs collected from antral follicles (Edwards, 1962). Its clinical use started in the early nineties (Cha *et al*., 1991) however its outcomes (embryo production, implantation and pregnancy rates) remain lower than conventional ICSI. In standard IVM protocols, oocytes are obtained from minimally stimulated or unstimulated ovaries, without hCG ovulatory trigger. Because IVM is more “patient-friendly” than other ART treatments, it is usually offered to women at risk of ovarian hyperstimulation syndrome (OHSS) (i.e. PCO/PCOS patients). Recently, an optimized two-step IVM culture system (CAPA-IVM) showed that by promoting oocyte-cumulus communication and synchronization of oocyte’s nuclear and cytoplasmic competences, the rates of oocyte maturation, good quality embryos on day 3 and clinical pregnancy versus standard IVM improved (Sanchez *et al*., 2017; 2019; Vuong *et al*., 2020a).

Despite the efficiency gap, in terms of live birth rate (35% vs 43%, for CAPA-IVM and IVF, respectively) (Vuong *et al*., 2020b), the significant reduction in cost and burden of IVM (compared to IVF) would make CAPA-IVM the first-choice treatment for PCOS patients (Braam *et al*., 2021).

**Case Description:** This is a single center pilot study in 5 infertile PCO patients. Enrolment took place between May 2022 and February 2023. Before initiation, patients have accepted and signed the informed consent. Patients were stimulated with 3 doses of HMG (Menopur^®^), 150IU daily, starting on day 1 to 3 of the cycle. Following 42-44h of the last injection, oocytes were retrieved by using a single-lumen 19G needle (Kitazato). COCs were handled and cultured similar to what has been reported previously (Sanchez *et al*., 2019). Oocyte maturation was assessed at 30h. MII oocytes were subjected to ICSI. In total, 55 Cumulus Oocytes Complexes (COC) were retrieved (mean: 11 COCs). MII and fertilization rates were 69.8%±15.4% and 68.3%±15.2%, respectively (mean ± SD). 3 out of 5 patients had at least 1 usable blastocyst that was/were vitrified to be transferred in a deferred cycle. Out of those 3 patients, one transfer has been performed, leading to our first pregnancy following a CAPA-IVM procedure. At the date of submission of this abstract, the patient is at 23-week gestation.

**Conclusion:** Oocyte In Vitro Maturation requires minimal (or no) ovarian stimulation prior to oocyte retrieval which makes it a simplified / patient-friendly / low-cost ART treatment. In the current pilot study, the effectiveness of CAPA-IVM at producing transferable embryos was explored. We conclude that CAPA-IVM is a promising technology that offers the possibility of low-cost ART treatment with a reasonable success rate.

**REFERENCES**

Braam SC, Ho VNA, Pham TD, Mol BW, van Wely M, Vuong LN. In-vitro maturation versus IVF: a cost-effectiveness analysis. Reprod Biomed Online. 2021;42:143-9. PMID: 33132059 DOI: 10.1016/j.rbmo.2020.09.022

Cha KY, Koo JJ, Ko JJ, Choi DH, Han SY, Yoon TK. Pregnancy after in vitro fertilization of human follicular oocytes collected from nonstimulated cycles, their culture in vitro and their transfer in a donor oocyte program. Fertil Steril. 1991;55:109-13. DOI: 10.1016/S0015-0282(16)54068-0

Edwards RG. Maturation in vitro of mouse, sheep, cow, pig, rhesus monkey and human ovarian oocytes. Nature. 1965;208:349-51. PMID: 4957259 DOI: 10.1038/208349a0

Sánchez F, Lolicato F, Romero S, De Vos M, Van Ranst H, Verheyen G, Anckaert E, Smitz JEJ. An improved IVM method for cumulus-oocyte complexes from small follicles in polycystic ovary syndrome patients enhances oocyte competence and embryo yield. Hum Reprod. 2017;32:2056-68. PMID: 28938744 DOI: 10.1093/humrep/dex262

Sanchez F, Le AH, Ho VNA, Romero S, Van Ranst H, De Vos M, Gilchrist RB, Ho TM, Vuong LN, Smitz J. Biphasic in vitro maturation (CAPA-IVM) specifically improves the developmental capacity of oocytes from small antral follicles. J Assist Reprod Genet. 2019;36:2135-44. PMID: 31399916 DOI: 10.1007/s10815-019-01551-5

Vuong LN, Le AH, Ho VNA, Pham TD, Sanchez F, Romero S, De Vos M, Ho TM, Gilchrist RB, Smitz J. Live births after oocyte in vitro maturation with a prematuration step in women with polycystic ovary syndrome. J Assist Reprod Genet. 2020a;37:347-57. DOI: 10.1007/s10815-019-01677-6

Vuong LN, Ho VNA, Ho TM, Dang VQ, Phung TH, Giang NH, Le AH, Pham TD, Wang R, Smitz J, Gilchrist RB, Norman RJ, Mol BW. In-vitro maturation of oocytes versus conventional IVF in women with infertility and a high antral follicle count: a randomized non-inferiority controlled trial. Hum Reprod. 2020b;35:2537-47. DOI: 10.1093/humrep/deaa240

**P-21. Off-label use of transdermal estrogen (Lenzetto^®^) during the COVID-19 pandemic: An experience at a human reproduction center in northern Colombia**

**Yeira López-Lora^1^, Felipe Vergara^1^, Carmen Vélez^1^, Imgard Amaya^1^, Isrrael Díaz-Yunez^2^, Alfredo Gómez^1^, Parra Guido^1^**

^1^Instituto de Reproducción Humana Procrear, Barranquilla, Colombia 080002.

^2^Centro de imágenes diagnósticas y terapéuticas CEDIUL, Barranquilla, Colombia 080002.

**Introduction:** COVID-19 and the primary effects of an outbreak are the direct consequences of the epidemic, however, the secondary effects of shortages are immediate consequences of the lack of access and services in the healthcare sector, assisted reproduction centers were not insusceptible to this scarcity, having to resort, according to prior knowledge, to medications that could substitute for this shortage, such as in the medication and replacement of endometrial preparation. The mains aim of this study was to evaluate the results of endometrial preparation and pregnancy rate in ART cycles, using transdermal estradiol hemihydrate (Lenzetto^®^) via a puff in post-thaw embryo transfer.

**Material and Methods:** Between June 2020 and March 2021, 57 patients attended a private human reproduction center to begin their endometrial preparation process. After the second day of the menstrual cycle or post-ACOs, the women received three applications of the transdermal medication puff-1.53 mg per dose in the morning and evening. An ultrasound control was carried out on the 9th day after starting estradiol hemihydrate. If the endometrium had a thickness greater than 7 mm and the dose was maintained, an additional dose of estradiol hemihydrate was added for four more days, and the endometrial thickness was reassessed. The application time was from 12 to 20 days. Embryo transfer and the use of vaginal progesterone were carried out according to the protocol.

**Results and Discussion:** The average age of the patients was 38.5±5.6 (range: 27-51). The average BMI was 24.3±4.6 kg/m^2^. The average administration dose was 4.9±0.9mg. No adverse events were reported. Of the 57 patients, 17 (29.8%) achieved a thickness ≥7mm with four applications, and 40 out of 57 (71.2%) achieved it with nine days of three puffs (29.8%). No patient showed dominant follicular development. The overall average endometrial thickness was 8.9±1.5 mm. One patient underwent embryo transfer on day 3, and the rest on day 5. The total number of transferred embryos was 66 (10 patients received two embryos, and 47 patients received one embryo). The pregnancy rate was 20 out of 57 (35.1%), biochemical pregnancy was 3/20 (15%), the abortion rate was 4 out of 20 (20%), and the live birth rate was 13 out of 20 (65%). No significant differences were found when comparing the values of Lenzetto application doses and endometrial thickness, as well as the number of applications in patients with and without pregnancy (3.2±0.5 *vs*. 3.1±0.6, respectively; *p*=0.79). There was no association between the administered dose and the occurrence of abortions (three in patients with three applications, and one with four applications).

**Conclusion:** The administration of 3-4 doses is suitable to achieve a thickness ≥ 7mm and a trilaminar morphology of the endometrium. The preparation scheme using transdermal estradiol hemihydrate (puff) may be an effective alternative method for endometrial preparation. Its safety and effectiveness should be evaluated in prospective studies that allow us to conclude that its routine use would be indicated, not only in times of shortage like those experienced in our country due to the effects of the COVID-19 pandemic.

**P-23. Fertility awareness in women with higher education**

**Paola Tamara Condea, Magdalena Decia, Gabriel de la Fuente, Dana Kimelman**

**Introduction:** The main objective of this study is to assess fertility awareness and knowledge about fertility care within higher education women in Uruguay, being the first study in our country to evaluate this specific population. Uruguay has a net reproduction rate of 0.95, if there are no changes in fertility, mortality, and migration, it will lead to a decrease in population. Since 1970, the fertility rate (live births / 1000 women per year) has fallen by almost 20%, with a population growth rate of 0.49% (GBD 2017 Population and Fertility Collaborators, 2018). This demographic change has been the result of a public policy trying to reduce teenage pregnancy but mainly it has been a consequence of women delaying their first pregnancy. Women with higher education have their first pregnancy 6 years later (Nathan, 2015). than the average and are expected to make an informed decision regarding their reproductive project.

**Materials and Methods:** This is a descriptive study. The target population of our study was Uruguayan women with complete or incomplete higher education. An anonymous online survey was conducted through social networks, google.com/forms was used for it. It was online a week. Questions were multiple choices for recording sample data and true or false questions to evaluate knowledge, using the Cardiff Fertility knowledges scale (Bunting *et al*., 2013) and adding questions focused on our population with a total of 27 questions (Table 1).

**Results:** A total of 1233 respondents were obtained. Of these, 62.6% were women over 35 years old and only 6.8% were under 25. 81.2% had a public tertiary education.47.9% educated in careers related to health. 54% have children with no significant difference between women linked or not to health. 47.3% were never counseled about their reproductive health, of whom 83.8% would have liked to be. From the responses to the questionnaire, it stands up that the group not linked to health marked the answer "I don’t know" on more occasions. Of these questions, the ones with higher error rates were: chances of pregnancy using their own oocytes in ART until menopause (60.1% answered yes) and the concept of associating a healthy life with improved fertility (76.5% answered wrong). 68% did not know how to respond to the frequency of infertility and 73.9% were unaware of the coverage of low-complexity treatments. Regarding the chances of pregnancy in women with primary amenorrhea 1/3 answered correct, 1/3 wrong and 1/3 did not know the answer. On the other hand, we highlight a correct response in more than 90%, ruling out semen production (96%) and erection (97.7%) as fertility indicators, and indicating that ART does not guarantee pregnancy (92.1%) (Table 1).

**Discussion:** Of the 27 questions that were asked, the most frequently missed questions were those that involved the coverage provided in our country, the incidence of infertility, concepts about women fertility especially about the ovarian reserve and the association of infertility with previous pathologies. There were no statistically significant differences in most of the questions between women linked to health or not. Our major concern is that x percent that an of our highly educated women believe that fertility is not affected by age.

**Conclusion:** We must emphasize fertility counseling in the gynecological consultation. Specially age impact in ovarian reserve. Explaining the concept of a limited number of oocytes and provide this concept to every patient that wills to receive this information. Further research is needed to evaluate providers’ knowledge of age’s impact on fertility. In other ways, we must reach the population and train physicians to be clear about Uruguayan law coverage so that they can refer patients at the right time.

**REFERENCES**

Bunting L, Tsibulsky I, Boivin J. Fertility knowledge and beliefs about fertility treatment: findings from the International Fertility Decision-making Study. Hum Reprod. 2013;28:385-97. DOI: 10.1093/humrep/des402

GBD 2017 Population and Fertility Collaborators. Population and fertility by age and sex for 195 countries and territories, 1950-2017: a systematic analysis for the Global Burden of Disease Study 2017. Lancet. 2018;392(10159):1995-2051. Erratum in: Lancet. 2019;393:e44.

Nathan M. La creciente heterogenicidad en la edad al primer hijo en el Uruguay: un analisis de las cohortes de 1951 a 1990. Notas Población. 2015;42:35-9. DOI: 10.18356/ecf4b5ea-es

**P-25. Successful pregnancy by Assisted Reproduction Techniques in a woman with husband seminal plasma allergy: When in vitro fertilization is a priority**

**Marai José B Santos^1,2^, Maria do Carmo B Souza^2^, Marcelo M Souza^2^, Roberto A Antunes^2^**

^1^ PRO-FEMININA-Barra Videohisteroscopia e Reprodução Humana.

^2^ FERTIPRAXIS Centro de Reprodução Humana.

**Introduction:** Hypersensitivity to human seminal plasma is a rare disease that is often misdiagnosed. Although this disorder is well described in the allergy and immunology literature, there are few cases in the gynecological literature. The clinical features of this condition may occur soon after intercourse or at a later stage and range from weak local reactions to life-threatening anaphylactic reactions.

**Case Report:** A 28-year-old Woman had an episode of allergy with systemic symptoms, that appeared within 30 minutes after intercourse. According to her this story began after three years of relationship with her only partner. The first sign was small eyelid erythema, which was reaching larger measurements with each intercourse. In a certain time she had to look for an emergency unit with dyspnea, labial and eyelid bilateral angioedema that required her to stay in the hospital. Due to the severity of her case, even though the doctors did not believe in the cause of the allergy, she was instructed to use condom uninterruptedly. As they wanted to achieve pregnancy, the allergist advised them about the possibility of gradual vaginal desensitization (Lavery *et al*., 2020). performed in a hospital environment, with staff and equipment available to treat potentially serious reactions, as anaphylaxis. There would be a need of repeated procedures with no certainty result. Besides, the state of desensitization is transient. The main criterion for the diagnosis of sperm allergy was the absence of symptoms when using condoms during intercourse. In addition, skin tests results were used to determine specific allergenic antigens of the seminal plasma (Allam *et al*., 2015). Sperm barriers are usually recommended to prevent allergic reactions in human seminal plasma-allergic patients. However, this was not an acceptable alternative for them. She refused artificial insemination with semen washing fearing anaphylaxis. The best option we could propose to them in order to be pregnant without having the slightest contact with the seminal fluid was then an IVF (ICSI) treatment. She underwent in vitro fertilization and the first fresh embryo transfer progressed with spontaneous abortion. After two months, a new transfer of one frozen blastocyst resulted in a live birth healthy baby.

**Discussion:** Symptoms of seminal fluid hypersensitivity (SFH) are extremely varied. In about 30% of the cases, the manifestations are local, but most have some type of systemic reaction (70%). Symptoms start immediately, most of them in the first 30 minutes of contact. It is mandatory to inform the patient that SFH is not a cause of infertility. After desensitization there is possibility of natural pregnancies. There is no consensus on the ideal approach to conception in these patients. There are reports of successful pregnancies with the use of artificial insemination but there remains the possibility of anaphylaxis (Resnick *et al*., 2004).

Conclusion: SFH is a disorder that has been attracting attention from the medical community. The rarity of case reports is most likely underdiagnosed, especially in cases of local manifestations. There is a need for a personalized approach and attention to the couple who have experiente anaphylaxis and who wish to have baby safely.

**REFERENCES**

Allam JP, Haidl G, Novak N. Spermaallergie [Semen allergy]. Hautarzt. 2015;66:919-23. German. PMID: 26490774 DOI: 10.1007/s00105-015-3710-1

Lavery WJ, Stevenson M, Bernstein JA. An Overview of Seminal Plasma Hypersensitivity and Approach to Treatment. J Allergy Clin Immunol Pract. 2020;8:2937-42. PMID: 33039013 DOI: 10.1016/j.jaip.2020.04.067

Resnick DJ, Hatzis DC, Kanganis P, Liccardi FL, Lee-Wong M, Bernstein JA. The approach to conception for women with seminal plasma protein hypersensitivity. Am J Reprod Immunol. 2004;52:42-4. PMID: 15214941 DOI: 10.1111/j.1600-0897.2004.00180.x

**P-24. *In vitro* fertilization with imported eggs: Two-year experience**

**Ricardo Azambuja^1^, Fabiana Mariani Wingert^1^, Marta Ribeiro Hentschke^1^, Victória Campos Dornelles^1^, Isadora Badalotti-Teloken^1^, Alvaro Petracco^1^, Mariangela Badalotti^1^**

^1^Fertilitat-Reproductive Medicine Center.

**Introduction:** As maternal age advances in present days, the interest in receiving donated oocytes is increasing by women seeking pregnancy. Literature shows similar live birth rates after in vitro Fertilization (IVF) techniques with heterologous eggs when compared to homologous eggs. The oocytes importation is growing in Brazil, being a new possibility for these patients after appropriate psychological and medical evaluation. Thus, the objective of the present study was to report an assisted reproduction clinic experience regarding the use of imported eggs to IVF.

**Material and Methods:** Retrospective, observational study performed at a reproductive medicine center. Data were collected between Jan/2021 and Jan/2023. Intracytoplasmic sperm injection (ICSI) was processed and the embryos were cultivated in a time-lapse incubator (Embryoscope^®^, Vitrolife^®^). A total of 118 couples imported oocytes for IVF and were included in this report. Variables were expressed in minimum and maximum (min-max), mean±standard deviation (SD) and n(%). Statistical description was made using SPS Statistics for Windows, Version 20.0.

**Results:** The male age was 42.61±7.18 years old. Regarding semen source, four couples got donated sperm, one had percutaneous epididymal sperm aspiration (PESA) and the other 112 were from ejaculate. One couple have not inseminated any oocyte due to 0% of oocyte survival rate. In total, nine couples opted for embryo biopsy (PGT-A). From those 26 that were biopsied, 13 were euploid (50%). A total of 180 embryo transfers were performed (frozen, n=59; fresh, n=121). The clinical pregnancy rate per transfer was 50.55% (91/180) (23 live births, 52 ongoing pregnancies, 15 miscarriages, and one ectopic); the cumulative clinical pregnancy rate was 81.98% (91/111). The index of live births plus pregnancy with more than 12 weeks per clinical pregnancy was 82.4% (75/91), and 41.7% (75/180) per transfer, and cumulative rate 75/111 (67.6%). The laboratory results are shown in Table 1.

**Discussion:** The present study aim was to report an experience regarding the use of imported eggs to IVF. Results showed oocyte survival rate in these two years of experience similar to previous studies (OSR 90.4% in Ana Cobo *et al*., 2015; 91.8% in Cao *et al*., 2009). Also, oocyte inseminated, fertilization, blastocyst and pregnancy rates are similar to homologous eggs used in IVF.

**Conclusion:** The use of imported oocytes from young donors has demonstrated to be an effective alternative for couples that are not able to conceive using their own genetic material.

**P-26. Educational Project: Sensitization course on patient treatment aimed at the staff of reproduction clinics**

**Rebeca Millán Delgadillo^1^**

^1^Universidad Anáhuac.

**Introduction:** Every year, more and more people are turning to fertility treatments in a quest to solve the obstacles that may arise to achieve a successful pregnancy. They are expensive treatments that by their nature generate emotions that impact the lives of patients who resort to them generating greater sensitivity, irritability and reactivity due to constant stress and uncertainty at each stage of the process. In the 7 years of experience I have in this field of medicine and psychology, I have detected the need for medical, administrative and any other staff to be trained to have an adequate emotional response when interacting with patients and due to their vulnerable emotional state, be an external regulator; this can be the big difference in the experience they have of their treatment. Based on the analysis of the historical, contextual and practical background is presented the proposal for a workshop on sensitization for the staff of reproduction clinics consisting of a 60-minute session for 5 consecutive days. The impact of this workshop will be assessed through a survey of patients about their experience interacting with staff.

